# Pharmacological Evaluation of Antidepressant-Like Effect of Genistein and Its Combination with Amitriptyline: An Acute and Chronic Study

**DOI:** 10.1155/2015/164943

**Published:** 2015-11-22

**Authors:** Gaurav Gupta, Tay Jia Jia, Lim Yee Woon, Dinesh Kumar Chellappan, Mayuren Candasamy, Kamal Dua

**Affiliations:** ^1^Department of Life Science, School of Pharmacy, International Medical University, Bukit Jalil, Kuala Lumpur 57000, Malaysia; ^2^School of Medicine and Public Health, University of Newcastle, Newcastle, NSW 2308, Australia; ^3^School of Biomedical Science, University of Newcastle, Newcastle, NSW 2308, Australia

## Abstract

The present study was designed to evaluate the acute and chronic antidepressant effect of genistein in combination with amitriptyline in mice. Animals were divided into six groups (*n* = 6) for treatment with water, genistein, or amitriptyline, either alone or in combination for ten days. Animals were subjected to locomotor activity testing; tail suspension test (TST); and forced swim test (FST) and immobility time was recorded on day one and day ten. Acute treatment of all treatment groups did not significantly reduce the immobility time (*p* > 0.05). Chronic treatment of combination of genistein (10 mg/kg) and amitriptyline (5 mg/kg and 10 mg/kg) significantly reduced the immobility time as compared to control group (*p* < 0.001) and was comparable to amitriptyline alone (10 mg/kg). However, no changes in anti-immobility activity in combination of subeffective doses of genistein (5 mg/kg) and amitriptyline (5 mg/kg) were observed. Genistein at its standard dose (10 mg/kg) rendered synergistic effects in combination with subeffective dose of amitriptyline (5 mg/kg) and additive effects in combination with therapeutic dose of amitriptyline (10 mg/kg).

## 1. Introduction

Depression is the most common affective disorders (defined as disorders of mood rather than disturbances of thought or cognition); it may range from very mild conditions, bordering on normality, to severe (psychotic) depression accompanied by hallucinations and delusions. The emotional symptoms of depression described by Diagnostic and Statistical Manual of Mental Disorders are lack of interest, sadness, guiltiness, and suicidal thoughts while lack of sleep, headaches, pain, sleep disorders, changes in appetite, gastrointestinal disorders, and changes in psychomotor function are the physical symptoms of depression. Around 6.3 to 15.7% of the world's population is estimated to suffer depression once in life according to World Health Organization International Consortium of Psychiatric Epidemiology (WHO-ICPE). Additionally, 7 to 12% in men and 20 to 25% in woman are the estimation of lifetime prevalence of major depression in adults [1, 2].

Tricyclic antidepressant like amitriptyline is chemically heterocyclic compounds used effectively for treating depression since it acts as a serotonin-norepinephrine reuptake inhibitor, thereby increasing the concentration of these transmitters in the synapse. Since many side effects due to chronic administration limit the therapeutic treatment, so it is necessary to unveil new targeted drugs with the claim of a more favourable tolerability and efficacy profile [3].

Natural compounds including genistein, a soy isoflavone, have been suggested in treatment of depression. It is a potential molecule as legumes are part of traditional diets in many regions of the world. However, studies conducted in patients with depression are limited. A randomised controlled trial has demonstrated that depression scores decrease significantly after 1 and 2 years of genistein treatment in postmenopausal women [4]. Animal models are also useful to study the antidepressant-like activity. Treatment of genistein 10 mg/kg for 14 days in ovariectomised rats has been shown to reduce the immobility time, the marker of depression in forced swim test [5]. Beside that, consumption of dietary genistein renders a wide range of potential health benefits including alleviation of menopausal symptoms, chemoprevention of breast and prostate cancers, and cardioprotective effect [6, 7].

In the present study, we aimed to investigate the effect of genistein in combination with amitriptyline administered acutely and chronically on animal behaviour in tail suspension test (TST) and forced swim test (FST) and locomotor activity testing in mice. We hypothesised that genistein would potentiate the antidepressant effect of amitriptyline. Ultimately, the combined therapy allows lower dose of amitriptyline to be employed and hence reduces its magnitude of side effects and may be effective in cases of treatment-resistant depression.

## 2. Materials and Methods

### 2.1. Animals

Adult male albino mice (BALB/c strain) weighing 20–30 g were used in this study. They were housed in group of 6 at a controlled temperature (25 ± 1°C) and humidity (50 ± 10%) with 12 h light/dark cycle (lights on at 7:00 am) with free access to standard pellet and water. Animals were used only once in each test. The experimental protocol was approved by the Laboratory Animal Care and Use Committee, International Medical University, Kuala Lumpur, Malaysia.

### 2.2. Drugs and Treatment

Amitriptyline (Sigma-Aldrich) and genistein (Sigma-Aldrich) were purchased from CHEMOLAB Supplies (Malaysia). All drugs were dissolved in reverse osmosis (RO) water and administered 1 hour before test. All test solutions were freshly prepared and administered orally in a volume of 10 mL/kg body weight for 10 days. Animals were randomly divided into 6 groups (*n* = 6) as follows. The doses were selected based on those reported in the literature.

All the mice were administered orally one hour before the locomotor activity testing; TST and FST were carried out on day one and day ten as follows:

 Group  1:  [control]: RO water (0.5 mL),
 Group  2:  [standard]: amitriptyline 10 mg/kg,
 Group  3:  [standard]: genistein 10 mg/kg,
 Group  4:  [test]: genistein 5 mg/kg + amitriptyline 5 mg/kg,
 Group  5:  [test]: genistein 10 mg/kg + amitriptyline 5 mg/kg,
 Group  6:  [test]: genistein 10 mg/kg + amitriptyline 10 mg/kg.

### 2.3. Locomotor Activity Testing

The locomotor activity was measured on innate pretreated mice by an actophotometer. Actophotometer functioned on photoelectric cells that were attached in circuit with a counter. When the ray of light dropping on the photocell was cut off by the animal, a count was noted. These cutoffs were calculated for a period of 10 min and the number was used as a degree of the locomotor activity of the animal [8].

### 2.4. Tail Suspension Test (TST)

The TST was conducted according to the method of Steru et al. [9]. Mice both acoustically and visually isolated were suspended by their tail in the TST apparatus (50 height × 45 width × 12 cm depths) on the first day and tenth day of drug administration. The duration of immobility was recorded for test period of 5 minutes. Mice were considered immobile when they hung passively and completely motionless.

### 2.5. Forced Swim Test (FST)

According to the FST method described by Porsolt et al., the mice were placed individually into 5 L glass beakers filled with 15 cm height of water. The water was changed frequently to eliminate fur, urine, and excrement after each test was done. When the mice remained floating in the water without struggling, making only minimum movements of its limbs necessary to keep its head above the water surface, they were considered to be immobile. This was classified as induced depression. The total duration of immobility was recorded during 5-minute test. The immobility period was calculated by subtracting total time (5 minutes) from time spent in escaping behaviour such as swimming and climbing. Swimming was defined as movements throughout the glass beaker and climbing was considered as upward-directed movements of forepaws by the side of glass beaker. Antidepressant drug treatment reduced the length of time the animals remain immobile and increased the escaping behaviour [10–12].

### 2.6. Statistical Analysis

All results were presented as mean ± SEM and ^*∗∗∗*^ *p* < 0.001, ^*∗∗*^ *p* < 0.01, and ^*∗*^ *p* < 0.05 were considered significant. Data were analyzed by one-way ANOVA, followed by Dunnett's post hoc test using Graph Pad Prism version 5.0.

## 3. Results

**Figure 1:**
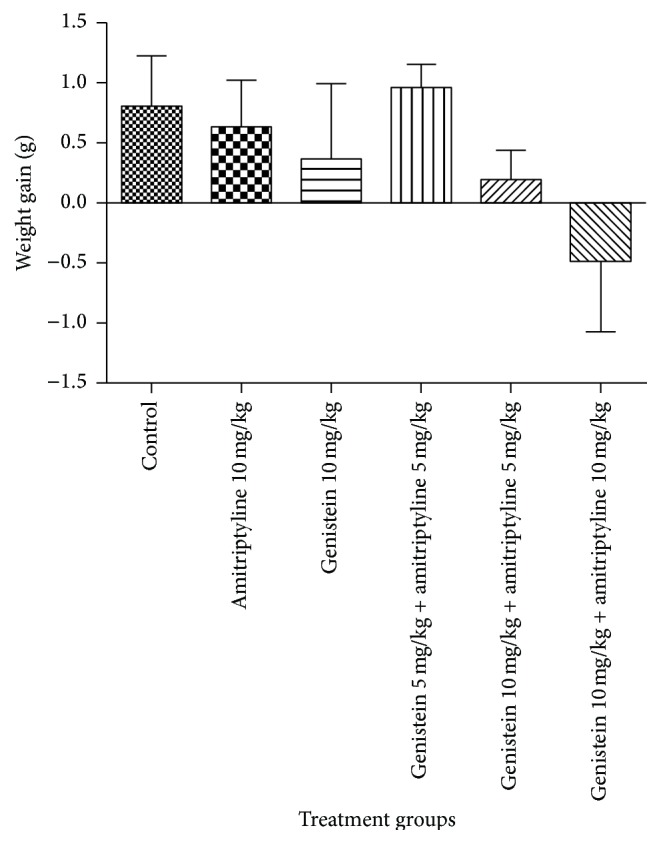
Effects of the standard drugs and their combined treatments on body weight gain in mice after 10 days. Values represent the mean ± SEM (*n* = 6). Data were analyzed with one-way ANOVA followed by Dunnett's post hoc test.

Body weight was evaluated before and after 10 days of treatment and weight gain is shown in Figure 1. Although weight loss was observed in the combination of genistein (10 mg/kg) and amitriptyline (10 mg/kg), there was no significant differences in body weight gain among all the treatment groups (*p* > 0.05).

In locomotor activity, as per Table 1, amitriptyline (10 mg/kg) shows a nonsignificant increase, whereas genistein significantly increases a locomotor activity alone and in combination with different doses of amitriptyline, respectively (*p* < 0.05; *p* < 0.01; and *p* < 0.001).

**Figure 2:**
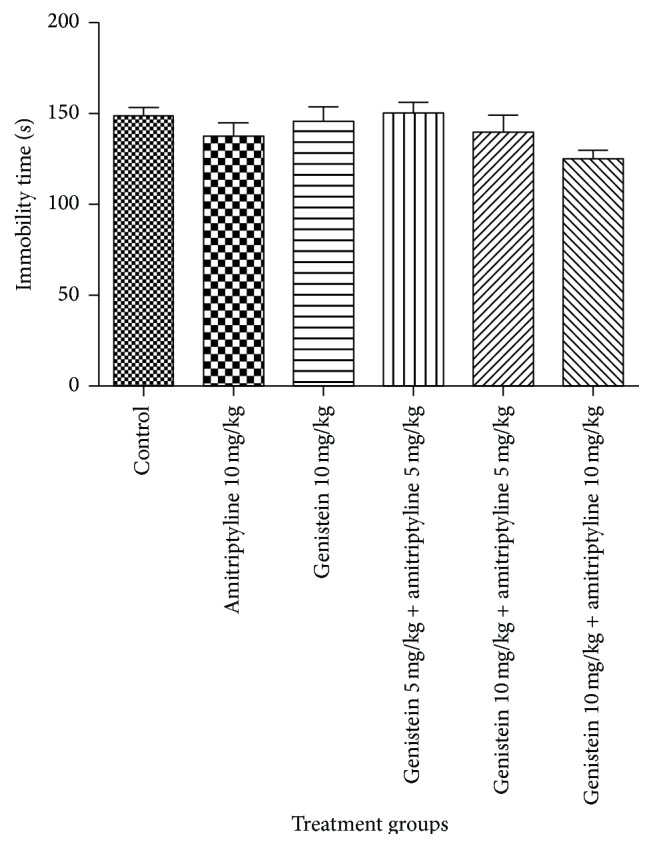
Effects of the standard drugs and their combined treatments on immobility time on 1st day. Values represent the mean ± SEM (*n* = 6). Data were analyzed with one-way ANOVA followed by Dunnett's post hoc test.

In TST, as shown in Figure 2, all treatment groups did not alter the total immobility time as compared to control group on the first day of experiment.

**Figure 3:**
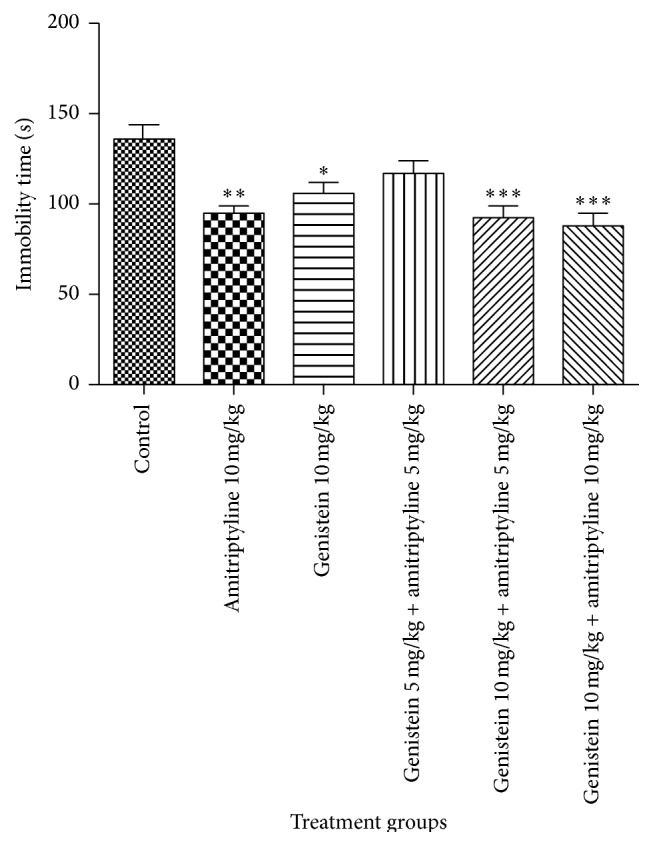
Effects of the standard drugs and their combined treatments on immobility time on 10th day. Values represent the mean ± SEM (*n* = 6). ^*∗∗∗*^ *p* < 0.001, ^*∗∗*^ *p* < 0.01, and ^*∗*^ *p* < 0.05 were considered significant as compared to control group. Data were analyzed with one-way ANOVA followed by Dunnett's post hoc test.

The behavioural effects after 10-day treatment are shown in Figure 3. On 10th day, individual treatments of amitriptyline (10 mg/kg) and genistein (10 mg/kg) produced significant decrease in immobility time as compared to control group (*p* < 0.05). The decrease in immobility time of genistein was comparable to amitriptyline. Coadministration of genistein (10 mg/kg) with amitriptyline (5 mg/kg and 10 mg/kg) significantly decreased the immobility time as compared to control group (*p* < 0.001) and were comparable to standard amitriptyline (10 mg/kg) and genistein (10 mg/kg) alone. However, the combination of genistein and amitriptyline at subeffective doses (5 mg/kg) did not significantly reduce the immobility time as compared to control group (*p* > 0.05).

**Figure 4:**
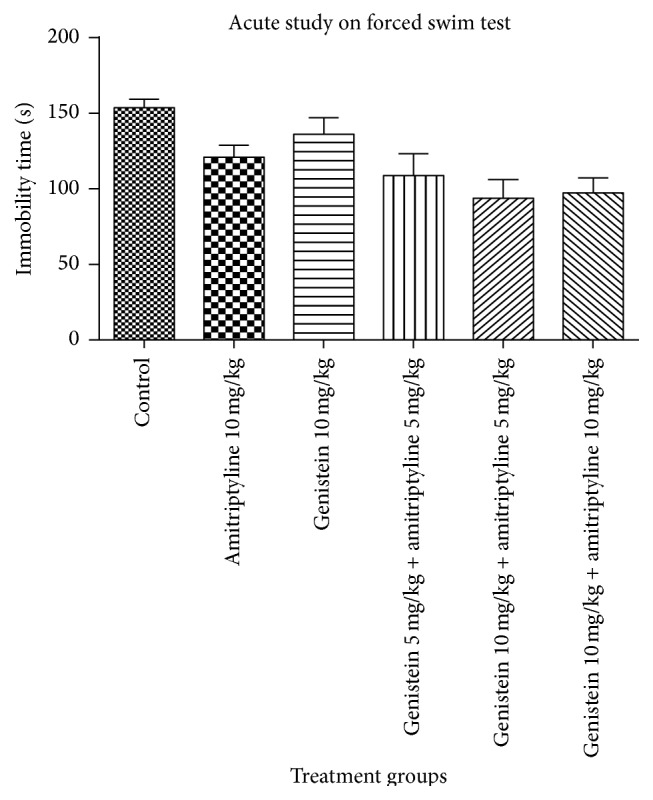
Immobility time (sec) in mice in forced swim test. Values represent the mean ± SEM (*n* = 6). All groups were compared with vehicle control (one-way ANOVA followed by Dunnett's test).

**Figure 5:**
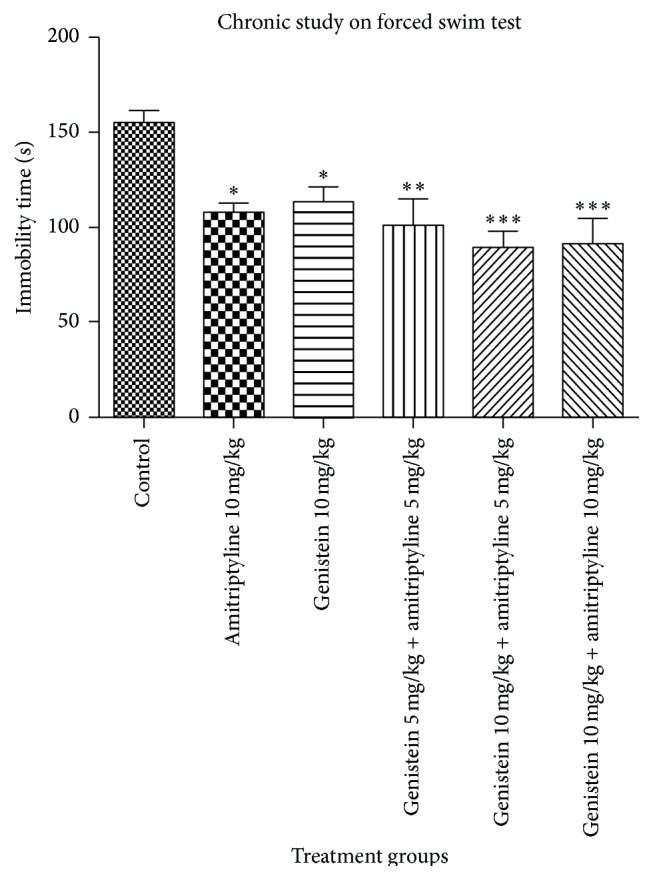
Immobility time (sec) in mice in forced swim test. Values represent the mean ± SEM (*n* = 6). ^*∗*^ *p* < 0.05, ^*∗∗*^ *p* < 0.01, and ^*∗∗∗*^ *p* < 0.001 were considered significant; all groups were compared with vehicle control (one-way ANOVA followed by Dunnett's test).

In FST, on day one, mean immobility period was reduced in animals treated with amitriptyline 10 mg/kg alone, genistein 10 mg/kg alone, and combined use of genistein 5 mg/kg with amitriptyline 5 mg/kg and genistein 10 mg/kg with amitriptyline 5 mg/kg and genistein 10 mg/kg with amitriptyline 10 mg/kg compared to control group but there was no statistically significant difference in duration of immobility in all groups of animals compared to animals in control group on first day, Figure 4. After ten days of administration, decrease in mean duration of immobility in all groups of animals was found to be statistically significant comparable to that of control groups, Figure 5. Genistein with dose of 10 mg/kg and amitriptyline with dose of 5 mg/kg in combination group showed most significant decrease in immobility period in FST as compared to control group on day ten (*p* < 0.001).

## 4. Discussion

Acute treatment of amitriptyline does not produce antidepressant effect similar to previous findings [13]. This may be due to delayed onset of action of antidepressant drugs observed in clinical settings. However, some studies have demonstrated that amitriptyline reduces the immobility time in rodents on the first day of behavioural test [14, 15]. Nonetheless, amitriptyline administered chronically exerts antidepressant activity. It is widely accepted that monoamines reuptake inhibition is crucial for its action. By blocking the serotonin and noradrenaline transporters, amitriptyline increases the neurotransmitters in the synapse and hence enhances neurotransmission [16]. Recent studies have reported that it activates fibroblast growth factor receptor signalling in glial cells which contribute to its antidepressant action [17]. Several mechanisms involved in the mediation of the antidepressant action after repeated treatment have been proposed. It is suggested that chronic treatment may enhance dopamine transmission in nucleus accumbens and also produce peptide of luteinizing hormone releasing hormone that modulates affective disorder [18–20].

Consistent with previous findings [5, 21], acute treatment of genistein does not exert antidepressant-like activity but repeated treatment shows antidepressant effect which is comparable to amitriptyline. Genistein has poor oral bioavailability. It is rapidly eliminated from plasma and excreted in the urine within 24 hours after single dose [6, 22, 23]. Hence, daily administration of genistein might increase plasma concentration of genistein but should not expect a dramatic increase in plasma level after 2-3 doses of treatment. A six-month randomised controlled trial has shown that plasma levels of genistein increase in first and sixth month with daily soy isoflavone administration [24].

Chronic genistein (10 mg/kg) in combination with therapeutic doses of amitriptyline (10 mg/kg) has significant antidepressant activity as compared to control group and is comparable to individual treatments. This suggests an additive effect at antidepressant dose of amitriptyline. On the other hand, genistein (10 mg/kg) in combination with subeffective doses of amitriptyline (5 mg/kg) administered chronically exerts antidepressant effect which is comparable to individual treatments, suggesting synergistic effect at subeffective dose of amitriptyline. Nevertheless, subeffective dose of genistein (5 mg/kg) fails to augment antidepressant effect of amitriptyline.

One of the side effects of tricyclic antidepressants is weight gain. In this study, we failed to demonstrate body weight gain in mice treated with amitriptyline. Studies in rats have revealed that there is no linkage between weight gain and amitriptyline treatment [25, 26]. From here, we cannot model the body weight changes of combination therapy in clinical setting. Additionally, genistein in agreement with previous report does not increase body weight in rats [5].

Genistein presents mainly in leguminous plants where 1 gram of soy protein contains 250 *µ*g of genistein [7]. Moreover, 10 mg/kg body weight of genistein for rats corresponds with 600 mg for a human [27]. The underlying mechanism of genistein in depression remains unclear. Genistein shares structural features with oestrogen-oestradiol-17*β* [7]. It can penetrate the blood-brain barrier after administration and bind differentially to *α* or *β* oestrogen receptors (OR) in the hippocampus [28]. Studies have concluded that antidepressant effect may involve OR *β* rather than OR *α* [29]. Furthermore, genistein may regulate serotonergic pathway under stressful conditions. In their study, genistein decreased serotonin turnover ratio (5-HIAA/5-HT), indicating serotonin level increased in hippocampus. Monoamine-oxidase A (MAO-A) is a mitochondrial enzyme involved in metabolism of monoamines such as dopamine and serotonin. MAO-A inhibitors are effective in treatment of depression. Hence genistein may regulate activity of MAO-A in brain [30]. Oxidative stresses are correlated with the pathophysiology of depression [31]. Therefore it suggests that antioxidant property of genistein may contribute to its antidepressant-like activity.

Importantly, genistein in moderation is well tolerated [32, 33]. Larger doses of genistein have been demonstrated to increase apoptosis and may be contraindicated in cancer regimens. In clinical trials, gastrointestinal adverse effects are the most common reason for treatment discontinuation. In addition, there is no change in endometrial thickness and breast density with daily administration of 54 mg of genistein for 3 years in postmenopausal women [32].

To our knowledge, the current study first time demonstrates the additive and synergistic effect of genistein in combination with antidepressant drug. Genistein (10 mg/kg) can be an adjunctive treatment with amitriptyline (10 mg/kg) for patients resistant to the conventional treatments. Additionally, genistein (10 mg/kg) in combination with subeffective dose of amitriptyline (5 mg/kg) may reduce the dose and thus the side effects of antidepressant drugs. This is important in cases of childhood depression or postnatal depression where safety of medication is being questioned.

Our results constitute further studies concerning the locomotor activity. Although previous studies have demonstrated that amitriptyline and genistein do not affect locomotor activity in rodents, locomotor activity shall be assessed to distinguish antidepressants from psychostimulants to prevent false positive results [34]. Further investigations, for instance, measurement of corticosterone, pharmacokinetic studies, and toxicology assessments, are recommended for better understanding of the observed phenomenon.

## 5. Conclusions

In summary, the present study supports the hypothesis that genistein enhanced the antidepressant effect of amitriptyline in tail suspension test. These findings might be important in the development of new treatment strategies and in the medical practice. The synergistic effect could mean a higher response for those with treatment-resistant depression and reduces the magnitude of side effects with low dose of antidepressant drugs.

## Figures and Tables

**Table 1:** Effects of the standard drugs and their combined treatments on locomotor activity.

Treatment (dose, mg/kg, p.o.)	Activity counts (s)
Normal control	165.36 ± 9.05
Amitriptyline 10 mg/kg	67.03 ± 5.38
Genistein 10 mg/kg	226.4 ± 12.35^c^
Genistein 5 mg/kg + amitriptyline 5 mg/kg	153.7 ± 13.27^b^
Genistein 10 mg/kg + amitriptyline 5 mg/kg	189.3 ± 10.14^c^
Genistein 10 mg/kg + amitriptyline 10 mg/kg	102.35 ± 14.12^a^

Values are expressed in mean ± SEM, where *n* = 6.

^a^ *p* < 0.05, compared with normal control group.

^b^ *p* < 0.01, compared with normal control group.

^c^ *p* < 0.001, compared with normal control group.

## References

[1] American Psychiatric Association (1994). *Diagnostic and Statistical Manual of Mental Disorders, (DSM-IV-R)*.

[2] Rihmer Z., Angst J., Sadock B. J., Sadocl V. A. (2005). Mood disorders: epidemiology. *Kaplan & Sadock's Comprehensive Textbook of Psychiatric*.

[3] Owens M. J., Morgan W. N., Plott S. J., Nemeroff C. B. (1997). Neurotransmitter receptor and transporter binding profile of antidepressants and their metabolites. *Journal of Pharmacology and Experimental Therapeutics*.

[4] Atteritano M., Mazzaferro S., Bitto A. (2014). Genistein effects on quality of life and depression symptoms in osteopenic postmenopausal women: a 2-year randomized, double-blind, controlled study. *Osteoporosis International*.

[5] Kageyama A., Sakakibara H., Zhou W. (2010). Genistein regulated serotonergic activity in the hippocampus of ovariectomized rats under forced swimming stress. *Bioscience, Biotechnology, and Biochemistry*.

[6] Kalaiselvan V., Kalaivani M., Vijayakumar A., Sureshkumar K., Venkateskumar K. (2010). Current knowledge and future direction of research on soy isoflavones as a therapeutic agents. *Pharmacognosy Reviews*.

[7] Dixon R. A., Ferreira D. (2002). Genistein. *Phytochemistry*.

[8] Kulkarni S. K. (2005). *Handbook of Experimental Pharmacology*.

[9] Steru L., Chermat R., Thierry B., Simon P. (1985). The tail suspension test: a new method for screening antidepressants in mice. *Psychopharmacology*.

[10] Porsolt R. D., Bertin A., Jalfre M. (1977). Behavioral despair in mice: a primary screening test for antidepressants. *Archives Internationales de Pharmacodynamie et de Therapie*.

[11] Detke M. J., Rickels M., Lucki I. (1995). Active behaviors in the rat forced swimming test differentially produced by serotonergic and noradrenergic antidepressants. *Psychopharmacology*.

[12] Borsini F., Meli A. (1988). Is the forced swimming test a suitable model for revealing antidepressant activity?. *Psychopharmacology*.

[13] Cryan J. F., Mombereau C., Vassout A. (2005). The tail suspension test as a model for assessing antidepressant activity: review of pharmacological and genetic studies in mice. *Neuroscience and Biobehavioral Reviews*.

[14] Enríquez-Castillo A., Alamilla J., Barral J. (2008). Differential effects of caffeine on the antidepressant-like effect of amitriptyline in female rat subpopulations with low and high immobility in the forced swimming test. *Physiology and Behavior*.

[15] Aithal S., Hooli T. V., Patil R., Varun H. V., Swetha E. S. (2014). Evaluation of antidepressant activity of topiramate in mice. *Asian Journal of Pharmaceutical and Clinical Research*.

[16] Leucht C., Huhn M., Leucht S. (2012). Amitriptyline versus placebo for major depressive disorder. *Cochrane Database of Systematic Reviews*.

[17] Hisaoka K., Tsuchioka M., Yano R. (2011). Tricyclic antidepressant amitriptyline activates fibroblast growth factor receptor signaling in glial cells: involvement in glial cell line-derived neurotrophic factor production. *The Journal of Biological Chemistry*.

[18] Borsini F., Nowakowska E., Pulvirenti L., Samanin R. (1985). Repeated treatment with amitriptyline reduces immobility in the behavioural ‘despair’ test in rats by activating dopaminergic and *β*-adrenergic mechanisms. *Journal of Pharmacy and Pharmacology*.

[19] Cervo L., Samanin R. (1988). Repeated treatment with imipramine and amitriptyline reduced the immobility of rats in the swimming test by enhancing dopamine mechanisms in the nucleus accumbens. *Journal of Pharmacy and Pharmacology*.

[20] Jain M. R., Subhedar N. K. (1993). Increase in number of LHRH neurones in septal-preoptic area of rats following chronic amitriptyline treatment: implication in antidepressant effect. *Brain Research*.

[21] Sapronov N. S., Kasakova S. B. (2008). Effects of synthetic and plant-derived selective modulators of estrogen receptors on depression-like behavior of female rats. *Bulletin of Experimental Biology and Medicine*.

[22] Kwon S. H., Kang M. J., Huh J. S. (2007). Comparison of oral bioavailability of genistein and genistin in rats. *International Journal of Pharmaceutics*.

[23] Busby M. G., Jeffcoat A. R., Bloedon L. T. (2002). Clinical characteristics and pharmacokinetics of purified soy isoflavones: single-dose administration to healthy men. *American Journal of Clinical Nutrition*.

[24] Gleason C. E., Carlsson C. M., Barnet J. H. (2009). A preliminary study of the safety, feasibility and cognitive efficacy of soy isoflavone supplements in older men and women. *Age and Ageing*.

[25] Nobrega J., Coscina D. V. (1987). Effects of chronic amitriptyline and desipramine on food intake and body weight in rats. *Pharmacology Biochemistry and Behavior*.

[26] Ranjbar S., Pai N. B., Deng C. (2013). The association of antidepressant medication and body weight gain. *Online Journal of Health and Allied Sciences*.

[27] Sakakibara H., Honda Y., Nakagawa S., Ashida H., Kanazawa K. (2003). Simultaneous determination of all polyphenols in vegetables, fruits, and teas. *Journal of Agricultural and Food Chemistry*.

[28] Tsai T.-H. (2005). Concurrent measurement of unbound genistein in the blood, brain and bile of anesthetized rats using microdialysis and its pharmacokinetic application. *Journal of Chromatography A*.

[29] Walf A. A., Frye C. A. (2008). Rapid and estrogen receptor beta mediated actions in the hippocampus mediate some functional effects of estrogen. *Steroids*.

[30] Hatano T., Fukuda T., Miyase T., Noro T., Okuda T. (1991). Phenolic constituents of licorice. III. Structures of glicoricone and licofuranone, and inhibitory effects of licorice constituents of monoamine oxidase. *Chemical and Pharmaceutical Bulletin*.

[31] Herken H., Gurel A., Selek S. (2007). Adenosine deaminase, nitric oxide, superoxide dismutase, and xanthine oxidase in patients with major depression: impact of antidepressant treatment. *Archives of Medical Research*.

[32] Marini H., Bitto A., Altavilla D. (2008). Breast safety and efficacy of genistein aglycone for postmenopausal bone loss: a follow-up study. *Journal of Clinical Endocrinology and Metabolism*.

[33] Evans M., Elliott J. G., Sharma P., Berman R., Guthrie N. (2011). The effect of synthetic genistein on menopause symptom management in healthy postmenopausal women: a multi-center, randomized, placebo-controlled study. *Maturitas*.

[34] Socała K., Nieoczym D., Wyska E., Poleszak E., Wlaź P. (2012). Sildenafil, a phosphodiesterase type 5 inhibitor, enhances the antidepressant activity of amitriptyline but not desipramine, in the forced swim test in mice. *Journal of Neural Transmission*.

